# Stability in Infant Rule‐Learning?

**DOI:** 10.1111/infa.70134

**Published:** 2026-11-04

**Authors:** Lisa Wagner, Stephanie Wermelinger, Virginia Hoffman, Moritz M. Daum

**Affiliations:** ^1^ Department of Psychology and Jacobs Center for Productive Youth Development Developmental Psychology: Infancy and Childhood University of Zurich Zurich Switzerland

**Keywords:** cognition, eye tracking, language, MB3, rule learning, stability

## Abstract

We tested the stability of infants' rule learning in an independent 6‐month follow‐up of infants originally assessed as part of ManyBabies3. Valid data were available from N=74 infants at each measurement occasion (T1: 5–12 months; T2: 11–18 months) and from N=66 infants for the longitudinal analyses. Infants completed a gaze‐contingent eye‐tracking adaptation of the ABA/ABB syllable‐triplet task. Rule learning was measured as a novelty preference (greater looking times in rule‐inconsistent than rule‐consistent trials). Bayesian analyses provided no evidence of rule learning at either measurement occasion and no clear evidence for longitudinal stability.

## 1 Stability in Infant Rule‐Learning?

Infants' generalization of simple repetition‐based relations has been tested most prominently with the syllable‐triplet paradigm (Marcus et al. 1999). In this paradigm, infants are familiarized with trisyllabic pseudowords following either an ABA pattern (e.g., *ga ti ga*) or an ABB pattern (e.g., *ga ti ti*) and are subsequently tested with novel pseudowords that follow or violate the familiarized rule. However, a meta‐analytic review suggests that evidence for robust generalization is mixed across studies and methodologies (Rabagliati et al. 2019). In line with this, a close, multi‐center replication found no reliable preference for rule‐inconsistent test items (Geambau et al. 2023), raising questions about the robustness of the original effect.

Rule learning has been linked to later language development, making early task performance a potentially informative individual‐differences measure (Bettoni et al. 2020). Such interpretations require longitudinal stability, because group‐level effects do not necessarily imply reliable individual differences. From a developmental perspective, one key question is whether performance *begins to stabilize* over time rather than being fully stable early in life. Here, *emerging stabilization* refers to increasingly stronger associations between infants' novelty‐preference scores across measurement occasions with age. This question is particularly important because infant measures of attention, information processing, and preference often show limited longitudinal or test–retest stability (Byers‐Heinlein et al. 2022; Cristia et al. 2016; Hendry et al. 2019). Weak stability in ABA/ABB task performance would therefore challenge the use of this task as a developmental marker of individual differences.

We tested longitudinal stability and possible emerging stabilization in a gaze‐contingent eye‐tracking adaptation of the ABA/ABB rule‐learning paradigm. We operationalized rule learning as a novelty preference, that is, greater looking to rule‐inconsistent than rule‐consistent trials. Infants were assessed twice, approximately 6 months apart, to examine the research questions of (1) whether novelty preference was present at the second measurement occasion, (2) whether individual differences were longitudinally stable, and (3) whether longitudinal stability increased with age. Data at the first measurement occassion (T1) were collected as part of a broader multi‐lab project (ManyBabies3; Visser et al. 2026). The second measurement (T2) was collected independently at our site as a longitudinal follow‐up and is not part of the ManyBabies3 dataset included in the respective Registered Report. The 6‐month interval was selected to assess developmental stability across a period of substantial developmental change rather than short‐term test‐retest reliability.

## 2 Methods

We report how we determined our sample size, all data exclusions (if any), all manipulations, and all measures in the study. We preregistered the study (https://osf.io/u2qkr) and made the data collected and R codes available on the Open Science Framework (OSF, https://osf.io/en43f).

### 2.1 Participants

As part of ManyBabies3 (Visser et al. 2026), our lab assessed N=86 infants aged 5–11 months at T1. The same infants were invited to return 6 months later (± 2 weeks) for a second assessment using the same task. Most families (52.63%) had an income equal to or higher than the median household income of couples with children in Switzerland (CHF 12,614,‐). Caregivers were well‐educated, and 76.47% had a Bachelor's degree or higher. Because of calibration failure or fussiness (T1: n=11; T2: n=5) and seven families who did not return, the T1 and T2 analytic samples were not identical. Longitudinal analyses therefore included the n=66 infants with valid novelty‐preference scores at both measurement occasions. For a description of the final sample, see Table 1.

**TABLE 1:** Final sample.

		Age (Days)		
	n	M(SD)	Range	% Female
Measurement occasion 1 (T1)	74	260.62 (59.44)	[155, 364]	51.70
Measurement occasion 2 (T2)	74	444.95 (58.11)	[337, 546]	51.35
Longitudinal analysis (at T1)	66	256.55 (57.29)	[155, 362]	53.03
Longitudinal analysis (at T2)	66	442.09 (57.96)	[337, 546]	53.03

### 2.2 Procedure

Data were collected from March to June 2023 for T1 and from September to December 2023 for T2. T1 followed the ManyBabies3 protocol; data for T2 were collected as a local follow‐up using the same task and equipment, but not as part of the ManyBabies3 coordinated data collection. Participants were recruited from the research unit's participant database (via birth records) after caregivers registered interest in participation. Infants received a small gift (approximately CHF 5) and a participation certificate. The study was approved by the ethics committee of the UZH Faculty of Arts and Social Sciences and conducted in accordance with Declaration of Helsinki.

Both appointments began in the lab's playroom, where caregivers were briefed and signed informed consent (only at T1). After a warm‐up period, families relocated to the study room for the rule‐learning task, presented on a 17‐inch monitor (800 × 600 px) using an EyeLink 1000Plus eye tracker (SR Research, 500 Hz sample rate). Infants, seated approximately 55 cm from the screen on their caregiver's lap, viewed full‐screen stimuli (34 × 28 visual degrees). A three‐point calibration with an animated target was conducted without re‐calibration, consistent with the ManyBabies3 procedure. After the rule‐learning task, families also completed a caregiver‐infant interaction task and caregiver questionnaires, which were not analysed here.

### 2.3 Measures

Infants' rule learning was measured using the ManyBabies3 adaptation of the Marcus et al. (1999) task (for details on task development, see Visser et al. 2026). During familiarization, infants viewed a colored checkerboard while listening to an approximately two‐minute stream of trisyllabic pseudowords separated by brief pauses. The stream followed either an ABA (e.g., *ga ti ga*) or ABB (e.g., *ga ti ti*) repetition pattern. Infants were randomly assigned to one of the two rule types (ABA or ABB), which remained the same across measurement occasions while the specific speech stimulus used for familiarization changed.

Following the familiarization phase, infants completed 12 test trials. During these trials, the colored checkerboard remained visible, and infants heard novel pseudowords constructed from syllables not presented during familiarization. The checkerboard served as a constant visual fixation stimulus while the auditory stimuli were presented. Half of the test trials followed the familiarized rule (consistent trials), whereas the other half violated it (inconsistent trials). Test trials were gaze contingent: Each trial began once infants fixated a centrally presented attention‐getter and ended after two seconds of continuous looking away or after the 15‐s auditory sequence. Infants' responses were quantified as the summed fixation duration during each test trial.

Rule learning was operationalized as differential looking toward consistent and inconsistent test trials. Looking time was defined as the summed fixation duration per trial. For each infant, we log‐transformed looking duration for each test trial and calculated the mean log‐transformed looking duration separately for consistent and inconsistent trials. We then calculated a proportional novelty‐preference score by dividing the mean log‐transformed looking duration for inconsistent trials by the sum of the mean log‐transformed looking durations for consistent and inconsistent trials. Scores above 0.50 indicate longer looking toward inconsistent (novel) test trials, whereas scores below 0.50 indicate longer looking toward consistent trials.

### 2.4 Analyses Plan

We pre‐processed the eye‐tracking data using the Data Viewer software (SR Research) using the default parameters. We excluded trials in which infants were too fussy, caregivers interfered (e.g., pointed to the screen), or the eye tracker could not measure their gaze (T1: 6.00%, T2: 1.28% of all trials). Following the ManyBabies3 analysis guidelines, we analysed trials in which children looked at the screen for at least 1 s. Children needed to have at least two valid test trials, including at least one consistent and one inconsistent trial, to be included in the analyses.

We deviate from the preregistered analyses in three ways. First, we only report novelty preference at each measurement occasion and its longitudinal stability. Two preregistered predictor analyses were not pursued because they presupposed a group‐level novelty effect that was absent in ManyBabies3 (Visser et al. 2026). Second, rather than directly comparing trial‐level looking times to consistent and inconsistent trials, as originally planned, we tested whether infant‐level novelty‐preference scores differed from 0.50 at each measurement occasion. Longitudinal stability required a directly comparable infant‐level outcome at both measurement occasions; therefore, novelty‐preference scores were used as the primary outcome measure. Third, whereas the preregistration specified frequentist models, we implemented the analyses in a Bayesian framework to quantify the direction and uncertainty of effects in the modest longitudinal sample. The longitudinal model included infants with valid novelty‐preference scores at both measurement occasions.

We tested our research questions with Bayesian generalized linear models (“Gaussian” family). To address our first research question, we estimated novelty preference at each measurement occasion relative to 0.50; values above 0.50 indicate longer looking toward inconsistent trials. To address our second and third research questions, we predicted T2 novelty preference from T1 novelty preference, age at T2, and their interaction. A positive effect of T1 novelty preference indicates longitudinal stability, whereas a positive interaction between T1 novelty preference and age indicates emerging stabilization, that is, an age‐related increase in longitudinal stability. Predictors were z‐standardised. Models were fitted in the brms package (version 2.22.0; Bürkner (2017)) with six chains of 6000 iterations each (3000 warm‐up). Following the ManyBabies3 findings, we expected no effects of the predictors on looking time and therefore specified normally distributed priors (μ=0, σ=1) for T1 novelty preference, age, and their interaction. Normally distributed priors were used for all other parameters. We report posterior median coefficients, 89% credible intervals (CI), and probability of direction (pd), considering effects with pd>97% as likely to exist (Makowski et al. 2019).

## 3 Results

Mean novelty‐preference scores were close to 0.50 at both T1 (M=0.499, SD=0.068; n=74) and T2 (M=0.506, SD=0.068; n=74; see Figure 1). Bayesian estimation indicated that neither mean differed credibly from 0.50 (T1: Median = 0.00, 89% CI [−.01, 0.01], pd=54.94%; T2: Median = 0.01, 89% CI [−.01, 0.02], pd=78.59%; Table 2). Thus, infants showed no reliable preference for rule‐inconsistent over rule‐consistent test trials at either T1 or T2 (see Figure A1).

**FIGURE 1:**
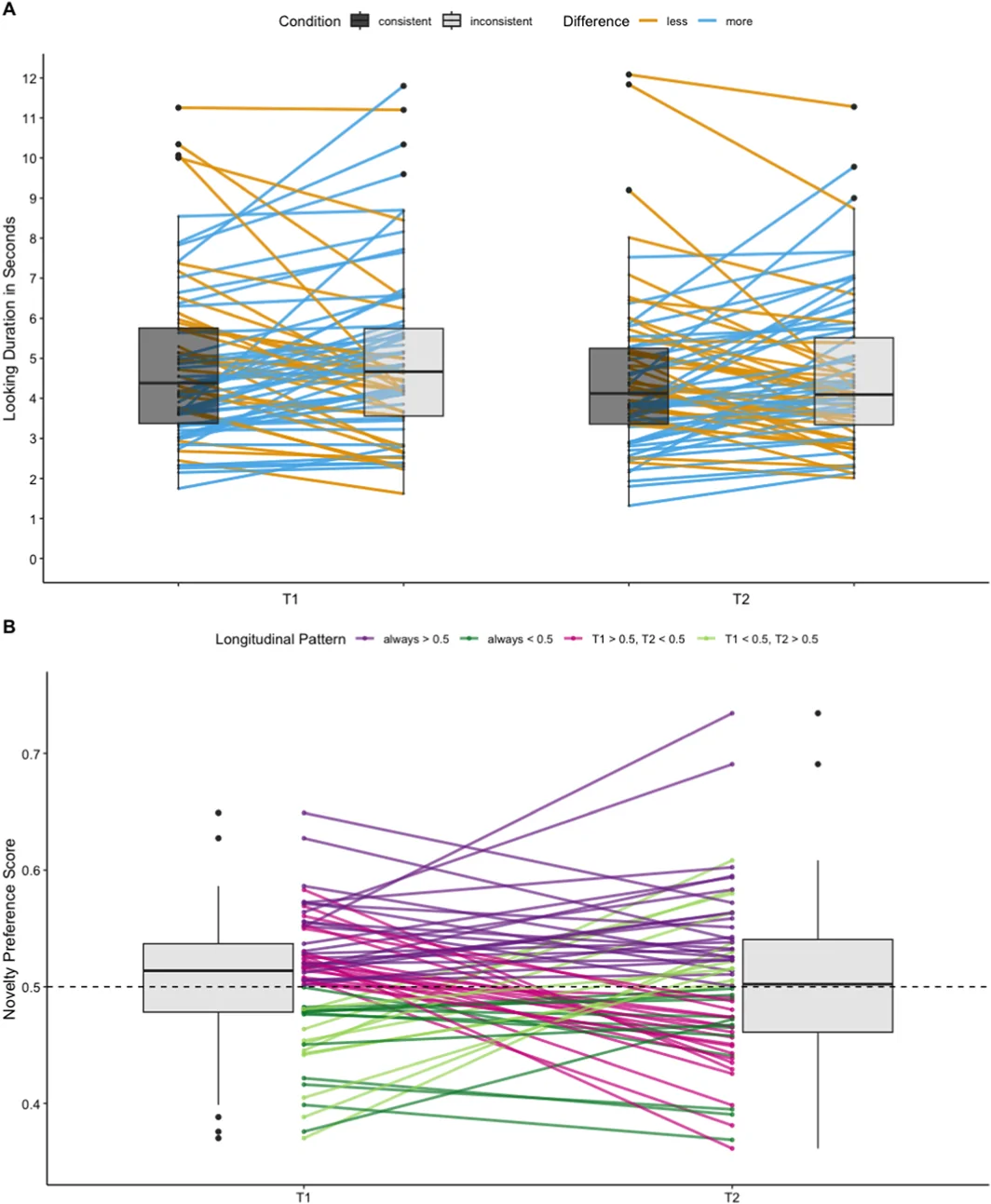
(A) Geometric mean looking duration in seconds to consistent (dark gray) and inconsistent (gray) test trials at the first (T1) and second (T2) measurement occasion. Lines connect each infant's geometric mean looking duration across trial conditions within a measurement occasion and indicate infants looking more (blue) or less (orange) to inconsistent than consistent trials. (B) Individual novelty‐preference scores at T1 and T2 connected by participant, illustrating longitudinal variability in novelty preference across measurement occasions.

**TABLE 2:** Results and details on computation.

Parameter	Median	89%CI	pd	Rhat	ESS
	Rule learning at T1				
Intercept	0.00	[−0.01, 0.01]	54.94	1.00	10,354
	Rule learning at T2				
Intercept	0.01	[−0.01, 0.02]	78.59	1.00	10,584
	Longitudinal analyses				
Intercept	0.50	[0.49, 0.52]	100	1.00	12,897
Novelty preference at T1	0.01	[0.00, 0.03]	91.44	1.00	13,877
Age	0.00	[−0.01, 0.02]	63.27	1.00	12,894
Novelty preference at T1 * age	0.02	[0.00, 0.03]	95.10	1.00	12,384

*Note:* pd = probability of direction in percent. Convergence of the chains is reported via the scale reduction factor (Rhat) and the stability of estimates via the effective sample size (ESS).

The longitudinal analyses provided very weak evidence that novelty‐preference scores at T1 positively predicted novelty‐preference scores at T2 (Median = 0.01, 89% CI [0.00, 0.03], pd=91.44%; Table 2). However, the estimated effect was very small and remained below our threshold for a likely effect. Likewise, the interaction between T1 novelty preference and age was very small and remained below the specified threshold (Median = 0.02, 89% CI [0.00, 0.03], pd=95.10%), providing no clear evidence of emerging stabilization. A robustness analysis using Bayesian trial‐level mixed‐effects similarly provided no clear evidence that looking duration differed between consistent and inconsistent trials or that this difference changed from T1 to T2 (see Appendix A).

## 4 Discussion

In this study, we addressed three related questions: whether infants showed evidence of rule learning, whether individual differences in rule learning remained stable across a 6‐month interval, and whether longitudinal stability increased with age. Using a gaze‐contingent eye‐tracking version of the ABB/ABA paradigm, we found no evidence of rule learning at either measurement occasion. Whereas the absence of a novelty preference at T2 extends the ManyBabies3 findings to an older age range, our primary contribution is that novelty‐preference scores showed little evidence of longitudinal stability across infancy. Individual differences in novelty preference showed no clear evidence of stability. Infants with a stronger novelty preference at T1 tended to have higher scores at T2, but the estimated association was very small and below our threshold for a likely effect. We therefore interpret this as, at best, very weak evidence of stability, rather than clear evidence for consistent individual differences over time.

Our findings converge with recent large‐scale and high‐fidelity replication efforts questioning the robustness of the original Marcus et al. (1999) finding. A close, multi‐center replication reported no reliable differences in looking between rule‐consistent and rule‐inconsistent test patterns (Geambau et al. 2023). Complementing this, the ManyBabies3 project (Visser et al. 2026) analysed data from 650 infants aged 5–12 months using a coordinated multi‐lab design. Across labs, ManyBabies3 found no systematic difference in looking between rule‐consistent and rule‐inconsistent test trials. Analyses also provided no evidence that task characteristics, participant variables, or analytical decisions recovered the original novelty preference. Our findings therefore extend ManyBabies3 by showing that (1) the absence of a novelty preference persisted when the same infants were re‐tested approximately 6 months later at an independently collected second measurement occasion (T2), which was not included in the ManyBabies3 dataset (Visser et al. 2026); (2) novelty‐preference scores showed little longitudinal stability across infancy; and (3) there was little evidence that longitudinal stability increased with age, indicating little evidence of emerging stabilization.

A central motivation for the study was whether ABA/ABB task performance might show *emerging stabilization* over early development. In other domains, early measures often show limited stability across infancy, including visual‐attention measures such as orienting and attentional control (Hendry et al. 2019), and looking‐time measures used across infant speech‐perception tasks (Cristia et al. 2016). Our results suggest that the ABA/ABB preference measure behaves similarly: Although infants varied in their novelty‐preference scores, these individual differences showed only weak longitudinal stability.

What do these findings imply for interpreting the ABA/ABB paradigm as a developmental measure? One possibility is that infants do not reliably form and generalize abstract repetition‐based relations under the conditions imposed by the canonical triplet task. Another possibility is that infants may be capable of such abstraction, but that this operationalization (i.e., decontextualized syllable triplets paired with a simple, non‐informative visual display) does not provide a sensitive or reliable assay, particularly for individual differences. In this view, performance may be dominated by transient state factors (e.g., attention, arousal, fatigue, moment‐to‐moment engagement) that vary across sessions, producing small or inconsistent group‐level effects and weak longitudinal continuity. This matters for developmental prediction: If task performance is not stable, it cannot serve as a strong individual‐differences marker for linking early rule learning to later outcomes (Bettoni et al. 2020).

More broadly, preference‐based infant measures often show limited test–retest reliability when used to infer stable individual differences (Cristia et al. 2016; Schreiner et al. 2024). Methodological recommendations, therefore, emphasize treating reliability as a primary design and reporting target (Byers‐Heinlein et al. 2022). The complementary ManyBabies3 Test–Retest project is currently investigating the short‐term test–retest reliability of the paradigm over a 1‐week interval (Exner et al. 2023). In contrast, the present study addresses developmental stability across a substantially longer 6‐month interval. The weak longitudinal association observed here is consistent with this broader pattern and suggests that, in its current form, the ABA/ABB triplet paradigm may have limited utility for measuring stable individual differences across infancy.

### 4.1 Limitations and Future Directions

Although substantial for a longitudinal infant eye‐tracking study, our sample limits precision for detecting very small effects, and conclusions may differ for other task variants, stimulus sets, or developmental periods. Moreover, because stability was assessed across only two measurement occasions, developmental change and measurement reliability cannot be disentangled. Consequently, weak longitudinal stability should not be interpreted as a direct estimate of measurement reliability. Future work should examine alternative rule‐learning paradigms, include additional longitudinal assessments, and evaluate converging measures that may provide more reliable indices of stable individual differences. The present findings help clarify what the ABA/ABB paradigm can and cannot measure.

## Author Contributions

**Lisa Wagner:** investigation, conceptualization, methodology, writing – original draft, writing – review and editing, supervision, data curation, project administration. **Stephanie Wermelinger:** conceptualization, investigation, methodology, formal analysis, visualization, writing – original draft, writing – review and editing, supervision, data curation, project administration. **Virginia Hoffman:** investigation, writing – review and editing. **Moritz M. Daum:** writing – review and editing, supervision, resources, project administration, conceptualization.

## Funding

The funding sources had no role in study design, analysis of data, interpretation of results, or writing of the report.

## Ethics Statement

The study was approved by the ethics committee of the Faculty for Arts and Social Sciences at the University of Zurich and conducted in accordance with Declaration of Helsinki.

## Conflicts of Interest

The authors declare no conflicts of interest.

## Figures and Tables

**FIGURE A1:**
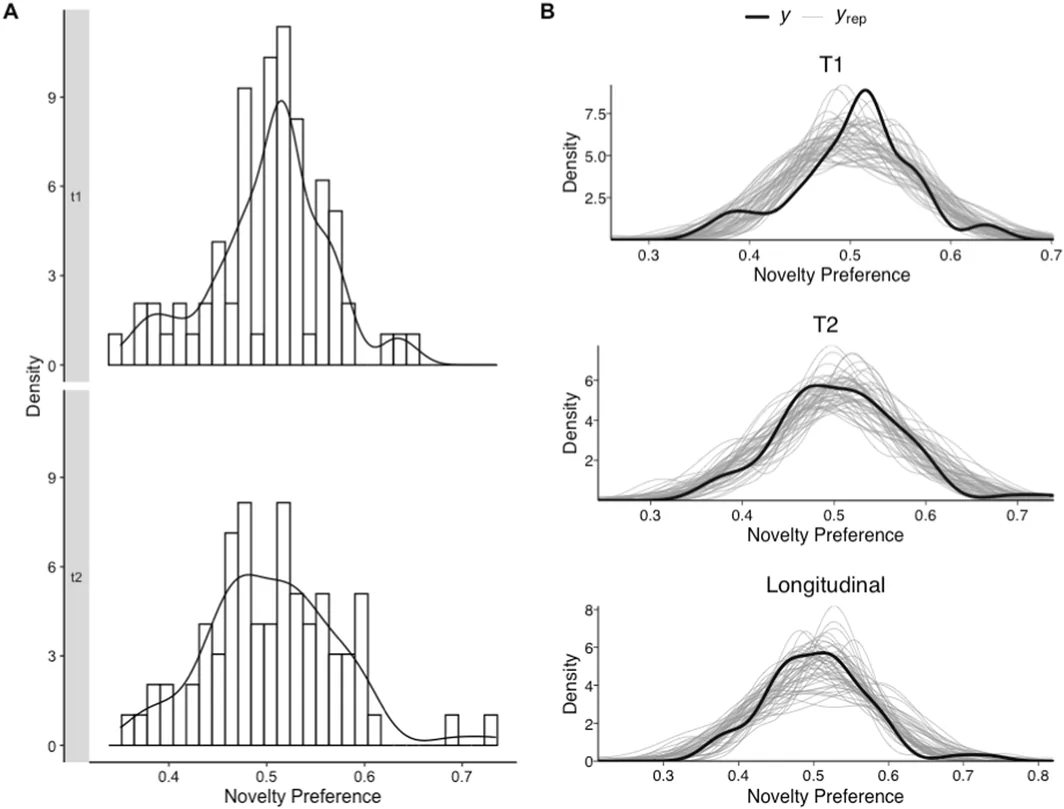
(A) Histogram of children's novelty preference at T1 and T2. (B) Posterior predictive check for the models, comparing the observed data (y) to simulated samples generated from the posterior predictive distribution $\left(y_{\mathrm{rep}}\right)$.

## Data Availability

The data that support the findings of this study are openly available in OSF at https://osf.io/en43f.

## Appendix A

### Trial‐Level Robustness Analysis

As a robustness analysis, we analysed trial‐level looking duration using a Bayesian skew‐normal mixed‐effects model. This analysis assessed whether conclusions were similar when modeling trial‐level looking times directly rather than infant‐level novelty‐preference scores. Fixed effects included familiarization rule, trial type, age, trial number, repetition pattern, measurement occasion, and their theoretically relevant interactions. Trial type was effect‐coded such that positive estimates indicated longer looking during inconsistent than consistent trials, and measurement occasion was effect‐coded with T1 = −1 and T2 = 1. Age and trial number were standardised. The model included participant‐specific random intercepts and uncorrelated random slopes for trial number, trial type, and their interaction. Normally distributed priors, N(0,1), were specified for all regression coefficients. The analysis provided no clear evidence that looking duration differed between inconsistent and consistent trials (Median = 0.01, 89% CI [−.06, 0.08], pd=60.83%). Likewise, there was no clear evidence that this difference changed between T1 and T2 (Median = 0.03, 89% CI [−.10, 0.17], pd=65.53%). Looking duration decreased across test trials (Median = −.42, 89% CI [−.52, −.33], pd=100%). All $\hat{R}$ values were 1.00 or 1.01, and the model sampled without divergent transitions.
